# Differential acceptance of a national digital health platform among community and frontline health workers in Cote d'Ivoire: a cross-sectional study

**DOI:** 10.3389/fdgth.2026.1785017

**Published:** 2026-05-04

**Authors:** Kadidiatou Raïssa Kourouma, Anatole Mian, Abdoul Gadiry Fadiga, Syntyche Bayo, Alimata Sow Diakité, Karin Källander, Eyram Adzra, Stéphane Yapi Yépié, Vincent Gnangui, Sébastien Gokou, Lama Karamoko, Ulrich Djololo, Bakary Kouamé, Ngondo Diomandé, Souleymane Yéo, Sékou Soumahoro, Françoise Kadja, Adama Pongathié, Franck Simon Bléhiri, William Yavo

**Affiliations:** 1 Institut National de Santé Publique, Abidjan, Cote d'Ivoire; 2 United Nations Children's Fund, Cote d'Ivoire Country Office, Abidjan, Cote d'Ivoire; 3 United Nations Children's Fund, New York, NY, United States; 4 United Nations Children's Fund WCARO, Dakar, Senegal; 5 Direction de la Santé Communautaire et de la Promotion de la Santé, Abidjan, Cote d'Ivoire; 6 Direction Régionale de la Santé du Poro, Korhogo, Cote d'Ivoire; 7 Direction de l'Information Sanitaire, Abidjan, Cote d'Ivoire; 8 Direction de l'Informatique et de la Santé Digitale, Abidjan, Cote d'Ivoire

**Keywords:** acceptance, community health workers, Cote d'Ivoire, digital health, frontline health workers, mHealth.ci, West Africa

## Abstract

**Introduction:**

Mobile-based digital health solutions are critical technologies that play a significant role in improving the quality of healthcare services. Cote d'Ivoire is digitizing its community-based health information system (CHIS), piloting the mHealth.ci that is composed of an eCHIS and other tools for community health workers (CHWs) and frontline health workers (FHWs) in the Poro health region. This study aimed to compare the determinants of the mHealth.ci acceptance between these two categories of end-users in Cote d'Ivoire.

**Methods:**

We conducted a cross-sectional analytic study from February to March 2025, in the five pilot health districts of the Poro Health Region, using a combined Technology Acceptance Model (TAM) and Louart S et al. framework. Key determinants included perceived usefulness, ease of use, advantages, disadvantages, social influence, personal emotion, context, attitude, intention to use, and actual use. A total of 280 trained end-users (80 FHWs and 200 CHWs) participated in the study. The data was analyzed with R.

**Results:**

Of 280 participants, 85% were male, with an average age of 42 ± 9 years. Significant socio-demographic differences existed between CHWs and FHWs (e.g., CHWs were older, less educated, and had less prior mobile app experience, *p* < 0.001). mHealth.ci was accepted by end-users, respectively 61.79% and 38.21% of the 280 end-users rated it as “totally acceptable” and “acceptable”. While perceived ease of use, attitude, personal emotion, intention to use, context, and perceived disadvantages showed no significant inter-group difference, CHWs perceived usefulness and advantages better (*p* < 0.001, *p* = 0.015 respectively), valued social influence more (*p* < 0.001), and reported greater actual use (*p* < 0.001). More FHWs needed time to adapt (*p* = 0.012) and were less satisfied (*p* = 0.007). CHWs reported insufficient training (*p* < 0.001) and paradoxically felt both confident and stressed using the mHealth.ci.

**Conclusion:**

The study provides important insights into mHealth.ci acceptance and emphasizes the necessity of adapting training and supervision approaches to the distinct needs of different end-user categories to foster successful adoption of mHealth.ci.

## 1 Introduction

Health system is based on a conceptual framework made of six pillars including health information system. The World Health Organization (WHO) defines health information system as a complex, multilevel system, aimed at producing health intelligence to inform decision-making, and encompassing data collection, analysis, health reporting, management and governance. Efficient and reliable health information systems can help improve patient outcomes, inform research, and influence policy- and decision-making (1).

The health information system also includes data collected at the community level. Indeed, in developing countries, the community-based health information system (CHIS) is a vital component of the community health system, as it assesses community-level healthcare service delivery and generates data for community health program planning, implementation, monitoring, and evaluation. CHIS promotes data-driven decision-making, by identifying priority interventions and programs, guiding resource allocation, and contributing to evidence-based policy development (2). Community health workers (CHWs) are increasingly acknowledged as a critical workforce to strengthening health systems and achieving sustainable development goals (3).

Several African countries are bolstering their CHIS through digitization to enhance community health programs and advance universal primary healthcare access. However, most CHIS systems in these countries are partner-driven, program-specific, and heavily reliant on donors' and partners' financial and technical support, as evidenced in the Democratic Republic of Congo, Egypt, Namibia, Kenya, and Burkina Faso (4–7). This reliance often leads to the creation of silos. Each partner brings their own rules, constraints, and priorities. Consequently, community health workers (CHWs) may find themselves burdened with multiple tools and devices, each tailored to the specific requirements of different partners. This fragmentation not only complicates the CHWs' tasks but also hinders the government's ability to consolidate data into a single, unified source of truth for community health information. The lack of integration and standardization across CHIS systems can impede effective decision-making, resource allocation, and the overall efficiency of health programs. The Ministry of Health, through the Directorate of Community Health and Health Promotion (DSCPS), created a national strategic plan for community health covering 2022–2025. This plan is aligned with the national health development plan for 2021–2025 and places a particular emphasis on the complete digitalization of health in Cote d'Ivoire. Although the country has had digital initiatives implemented by partners, these initiatives often remain isolated.

Thus, the DSCPS, with the support of UNICEF, identified the mHealth.ci digital platform as a solution adapted to Cote d'Ivoire's community health needs. The Ministry of Health is piloting mHealth.ci in five districts within the Poro health region. Information sessions and awareness campaigns have been held with targeted stakeholders in these districts. This digital platform is designed to be used by frontline health workers (FHW) and community health workers (CHW) (8). By providing a standardized and integrated platform, mHealth.ci aims to overcome the challenges created by the heavy dependency on partners and the resulting silos. It simplifies the tools and devices used by CHWs, ensuring a more streamlined and efficient approach to data collection and management. This, in turn, facilitates better decision-making, resource allocation, and overall effectiveness of community health programs.

Increasingly, mobile health is being adopted within health services and community health, representing a promising approach. Despite the adoption of digital platforms by several African nations, the majority—approximately 71%—still depend on paper-based systems for collecting community health information system data (9). In addition, factors such as infrastructural constraints, limited access to mobile phones, stable electricity sources, mobile network, hinder acceptance, and adoption of digital systems (5, 6, 10–12).

In Cote d'Ivoire, despite the shift in use of mHealth by CHWs and FHWs in the Poro Health region, little is known about acceptability and attitude among these end-users. Acceptability has become a key consideration in the design, evaluation, and implementation of healthcare interventions. Acceptability is a necessary but not sufficient condition for effectiveness of an intervention; however, successful implementation depends on the acceptability of the intervention (13).

Theoretical articles on acceptability in the literature distinguish between three main stages in acceptability: before the introduction (or before use) of an innovation, during its introduction (or initial use), and after its introduction (or sustained use) (13). In this study, we sought to compare the acceptance of mHealth.ci between CHWs and FHWs. By examining the determinants of acceptability for each group, we aim to gain insights into how different perceptions and experiences shape the acceptance of mHealth.ci.

## 2 mHealth.ci project description

The mHealth.ci project in Côte d'Ivoire represents a groundbreaking effort to significantly enhance both the accessibility and quality of community health data and services. This initiative specifically targets remote and vulnerable populations, leveraging the power of digital technology and mobile solutions to revolutionize healthcare delivery. At its core, the project aims to foster better collaboration and smoother interactions between CHWs and FHWs, thereby improving the quality of care provided by CHWs and ensuring the reliability of community health data. The mHealth.ci is designed to be adaptable and robust, functioning even in areas with low connectivity. It integrates seamlessly with the national health information system (DHIS2), providing real-time visibility on public health indicators. The platform includes modules for household registration, prenatal and postnatal follow-up, vaccination campaigns, and medical supply management, offering an integrated approach to community health needs.

### 2.1 Collaborative origins and keys stakeholders

This project is the result of a collaborative vision, primarily driven by the Ministry of Health through three directorates: Direction de la Santé Communautaire et de la Promotion de la Santé (DSCPS), Direction de l'Information Sanitaire (DIS), Direction de l'Informatique et de la Santé Digitale (DISD). UNICEF has been a crucial technical and financial supporter, playing a crucial role. Aligned with the 2021–2025 Community Health Strategic Plan, UNICEF commissioned the ICT startup Ticanalyse to develop the mHealth.ci application. The project is piloted in the Poro Health Region located in the northern part of Cote d'Ivoire. At the local level, the project's success hinges on the active engagement of prefectural and health authorities, community action officers, and the end-users (CHWs and FHWs).

### 2.2 Strategic deployment and training

The deployment of mHealth.ci followed a meticulously planned process to ensure its effective adoption. This began with essential preparatory activities, focusing on comprehensive stakeholder engagement and tailoring the application modules to the country specific context. This phase involved developing detailed specifications and conducting workshops to refine and validate the application's content. Following this, a cascading training program was rolled out. National experts received intensive training in August 2024. These experts then trained the FHWS who trained the CHWs in December 2024.

### 2.3 Pilot launch

The mHealth.ci project officially launched its pilot phase in December 2024, in the Poro health region. Each end-user received key equipment such as a smartphone, SIM card, and power bank. They had a two-week trial period for familiarizing with the platform before the full operational launch on January 8, 2025. The supervision of CHWs is a tiered system designed to ensure effective oversight and support. Frontline health workers are responsible for supervising CHWs. In turn, these FHWs are supervised by the district-level community activity supervisors. At the central level, the team of experts oversees the overall supervision of the entire system, conducting regular on-site visits to monitor operations and provide guidance.

## 3 Methods

### 3.1 Conceptual framework

We combined the Technology Acceptance Model (TAM) framework (14) and the Louart S et al. framework (15) to formulate hypotheses about the acceptance and use of the mHealth.ci among community health workers (CHWs) in the intervention area. The Technology Acceptance Model (TAM) is frequently used to explain and predict individuals' acceptance and behavior towards information technologies in healthcare and other sectors. TAM is widely used in studies exploring the acceptance and use of various health information technologies among patients and healthcare professionals. However, TAM is not applied to predict the acceptance of digital health tools by CHWs in Côte d'Ivoire. TAM consists of five major constructs: perceived usefulness, perceived ease of use, attitude, intention to use, actual use. The Louart S et al. framework includes seven constructs: perceived complexity, perceived disadvantages, personal emotions, social influence, compatibility, perceived benefits, and context. This framework is one of the few that considers context, personal emotion, and social influence.

In our study, the constructs selected were perceived usefulness, perceived ease of use, perceived advantages, perceived disadvantages, social influence, personal emotion, context, attitude, intention to use and actual use. We did not include perceived complexity because the study measured perceived ease of use which is often considered as its opposite. Information on perceived complexity are often captured when addressing perceived ease of use.

As for compatibility, this construction often overlaps with perceived usefulness and context. Furthermore, by measuring actual use, it allowed, beyond mere compatibility, for an assessment of how well the platform is integrated into routine practices.

**Figure 1:**
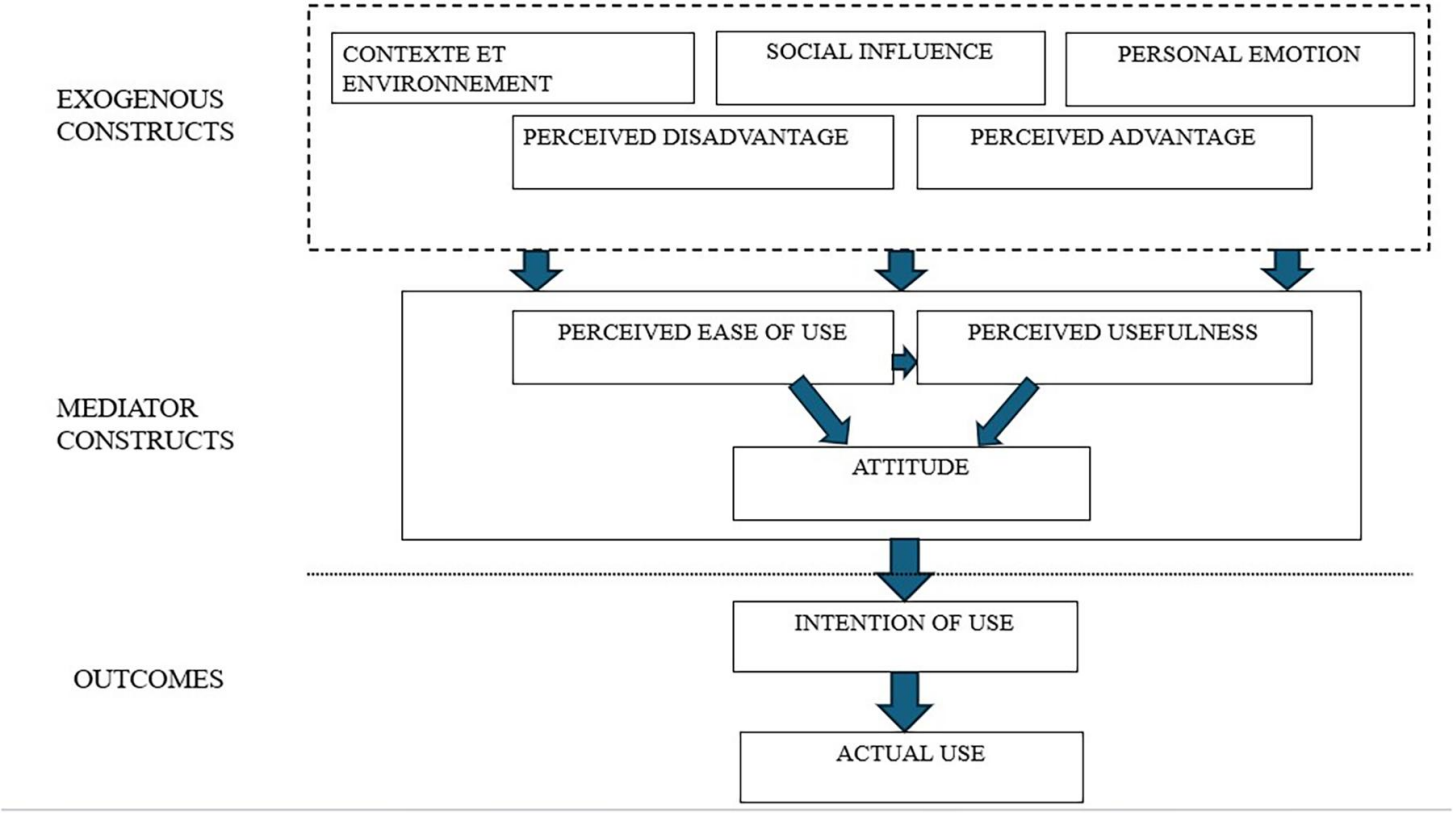
Integrated conceptual framework based on TAM and Louart S model.

The relationships among constructs are displayed in Figure 1.

Perceived usefulness (PU): this was defined as end-users' belief that mHealth.ci is useful.

Perceived ease of use (PEOU): refers to the degree to which a person believes that using the digital platform would be free of effort.

The perceived disadvantages (PR) are defined as the amount of effort, risk and cost perceived to be associated with innovation and its use.

Perceived advantages (PA) are the extent to which an innovation is perceived as bringing benefits.

Social influence (SN): is the extent to which other people's opinions influence one's degree of acceptability.

Personal emotion (PE): define this dimension as the emotions an individual feels about an innovation and its use.

Context and environment (FC): we described the “context” dimension as how the contextual environment (organizational, political, economic, social, etc.) influences the degree of acceptability of an innovation.

Attitude (ATT): this construct represents the user's overall positive or negative feelings about using technology.

Intention to use (ITU): is defined as the intention to continue using the mhealth.ci and recommend it to others.

Actual use (AU): it refers to the real and observable extent to which individuals employ the technology.

As illustrated in Figure 1, we hypothesized that user's acceptance of mHealth.ci is driven by a multi-layered interaction between cognitive, emotional, and contextual factors. PU and (PEOU) are primary drivers that influence the user's positive or negative attitude overall. PU, PEOU, and ATT are influenced by external factors like SN and FC. ATT determines ITU, which leads to AU and the platform's integration into routine practices. In your study, the user category (CHWs vs. FHWs) served as the primary independent grouping variable used to identify disparities in mHealth.ci acceptance across the entire conceptual framework.

While we adopted the Technology Acceptance Model (TAM) and Louart S frameworks to explore determinants of mHealth.ci use, it is important to acknowledge that “user acceptance” is not a static or sufficient condition for sustainable digital health implementation. In the broader literature on digital transformation and organizational change, acceptance is shaped by institutional readiness, supervision structures, training ecosystems, and broader system-level incentives. From this perspective, our use of acceptance constructs should be seen as a pragmatic entry point for capturing early user perceptions, which need to be interpreted within the larger context of health system change. This dual lens allows us to combine structured, measurable constructs with a critical awareness of their limitations.

### 3.2 Study design and setting

This was a cross-sectional analytic study conducted from February to March 2025, one month after the implementation of mHealth.ci. The study sites were the five pilot health districts of the Poro Health Region, located in the northern Cote d'Ivoire.

### 3.3 Study population and sampling

The study population were end-users trained in the use of mHealth.ci. Approximately, 600 end-users were trained across the five health districts. These end-users were community health workers (CHWs) and frontline health workers (nurses and midwives).

The sample size calculation was performed using Schwartz's formula:

$$n=\frac{Z^{2}\times p\times (1-p)}{e^{2}}$$

where:

Z is the z-score corresponding to the confidence level (e.g., 1.96 for a 95% confidence interval), p is the estimated proportion of the population (if unknown, 0.5 can be used to maximize the size), E is the acceptable margin of error (in our study, E = 0.05).

The minimum sample size was 235 end-users. In each of the five health districts selected, twelve health facilities were randomly selected from the health facilities involved in the project. In total, sixty health facilities were included in the study. Within each health facility, we aimed to interview six end-users, specifically one nurse, one midwife, and four CHWs.

Exclusion criteria included staff who were not trained in the use of mHealth.ci or who declined to participate.

### 3.4 Data collection

In each district, a team of two investigators collected data over a period of 11 days. In total, five teams, each composed of two investigators, conducted the data collection at a rate of two health facilities per day. Prior to any questionnaire administration, the informed consent of each respondent was obtained following a rigorous methodology. The questionnaire was administered face-to-face using a tablet on which the questionnaires had been digitized using ODK technology. Once collected, the data was sent to the ODK server.

The questionnaire was adapted from validated scales (TAM and Louart S et al). This questionnaire was based on the dimensions of the conceptual framework and consisted of thirty-three questions, including one general question regarding the level of acceptance. Each question was assigned a score ranging from 1 to 5 on a Likert scale (see Supplementary File 1). Regarding validity assessment, the questionnaire was reviewed and validated by a panel of experts of the National Public Health Institute to ensure the questions were relevant and accurately reflected the dimensions of the integrated conceptual framework. We also conducted in-room role-playing and a pre-test of the tool.

As for reliability, the investigators received three days of training on the overall survey procedures and the content of the data collection tool to harmonize data collection procedures. Furthermore, a data collector's manual was developed with a detailed description of standardized operational procedures to serve as a daily guide for data collection.

### 3.5 Data analysis

The collected data were exported to R version 4.4.0 for cleaning, coding, and analysis. Continuous data, such as scores, were described by their measures of central tendency (mean and standard deviation), and categorical data by their frequencies and percentages.

A descriptive analysis was performed to describe the sociodemographic variables of the end-users and to assess the differential acceptance between CHWs and FHWs using Chi square (Fisher's exact test) and Wilcoxon test. Effect sizes for Wilcoxon–Mann–Whitney tests were calculated using Cliff's Delta (*δ*), interpreted as small (|*δ*| = 0.147), medium (|*δ*| = 0.33), and large (|*δ*| = 0.474). The significance level was set at 0.05.

The Wilcoxon rank-sum test (Mann–Whitney U test) was chosen because it is an appropriate non-parametric test for comparing two independent groups (CHWs vs. FHWs) when the dependent variables (scores for each construct) are ordinal or continuous but not normally distributed. We have verified and noted that the key assumptions of the test independence of observations and that the distributions for both groups have a similar shape were met.

Before proceeding with the comparative analysis, the internal consistency of the scales was verified. The results indicated good reliability for PU (*α* = 0.80), acceptable reliability for four constructs such as ITU (*α* = 0.75) and AU (*α* = 0.76). Only FC showed poor reliability (*α* = 0.59). The four other construct indicated questionable reliability (see Supplementary File 2).

## 4 Results

### 4.1 Participants characteristics

As displayed in Table 1, the study involved a total of 280 end-users. Overall, most participants were CHW (71.43%), were male (85%) with a primary school level (43.93%). The average age was 42 ± 9 years. More than half of the participants (41,71%) had no prior experience with a mobile application. All results showed significant differences between the two groups regarding socio-demographic characteristics. The CHW group was, on average, older than the FHW group, less educated, and had less experience with mobile applications (*p* < 0.001).

**Table 1:** Participants characteristics (*N* = 280).

Variables	Total (*N* = 280)	CHWs (*N* = 200)	FHWs (*N* = 80)	*p*-value
Sex				**<0** **.** **001**
Female	15%	7.50%	33.75%	
Male	85%	92.50%	66.25%	
Education				**<0** **.** **001**
Primary school	43.93%	61.50%	0.00%	
High school	32.50%	38.50%	17.5%	
University	23.57%	0.00%	82.5%	
Years of experience				**<0** **.** **001**
<1 year	7.14%	1.50%	21.25%	
1 to 4 years	20.36%	15.50%	32.50%	
5 to 9 years	29.64%	28.00%	33.75%	
>10 years	42.86%	55.00%	12.50%	
Previous experience with a mobile application (application used in the healthcare field)				**<0** **.** **001**
No	59.29%	75.50%	18.75%	
Yes	41.71%	24.50%	81.25%	
Age				**<0** **.** **001**
Mean (SD)	42 (9)	45 (8)	35 (6)	

Values in bold indicate statistical significance at *p* < 0.05.

### 4.2 Level of acceptance among end-users

The mHealth.ci platform is widely accepted by end-users. Specifically, 61.79% of the 280 end-users rated the digital platform as “totally acceptable,” while the remaining 38.21% categorized it as “acceptable”. However, there is a statistically significant difference (*p* < 0.001) in the level of acceptance between CHWs and FHWs. CHWs saw the mHealth.ci as more “totally acceptable” compared to FHWs (Table 2).

**Table 2:** Level of acceptance per end-users' qualification.

Level of acceptance	Overall *N* = 280^a^	CHWs *N* = 200^a^	FHWs *N* = 80^a^	*p*-value^b^
				**<0.001**
4 = Acceptable	38.21%	31.0% (62/200)	56.25% (45/80)	
5 = Totally acceptable	61.79%	69.00% (138/200)	43.75% (35/80)	

Values in bold indicate statistical significance at *p* < 0.05.

^a^ % (n/N).

^b^ Pearson's Chi-squared test.

### 4.3 Analysis per constructs of the framework and qualification

There was no significant difference in PEOU, AT, PE, ITU, FC, PR between the two groups. Variability in responses by construct would be associated with qualification in terms of perceived usefulness (“(*p* < 0.001, Cliff's (δ) = 0.11 [95% CI: 0.05, 0.17], small effect)”), perceived advantages (“(*p* < 0.015, Cliff's (δ) = 0.09 [95% CI: 0.02, 0.17], small effect)”), social influence (“(*p* < 0.001, Cliff's (δ) = 0.19 [0.11, 0.26], small effect)”), actual use (“(*p* < 0.001, Cliff's (δ) = 0.17 [95% CI: 0.09, 0.24], small effect)”). The CHWs perceived the usefulness and benefits better than the FHWs and seemed to have integrated the platform more into their routine than the FHWs. Social influence, especially peer support, was important for CHWs compared to FHWs (Table 3).

**Table 3:** Average score by domain and end-user qualification (*N* = 280).

Domain	Overall *N* = 280	CHW *N* = 200	FHW *N* = 80	*p*-value^a^
PU_score				**<0**.**001**
Median (Min - Max)	4.00 (1.00–5.00)	4.00 (2.00–5.00)	4.00 (1.00–5.00)	
Mean (SD)	4.39 (0.55)	4.42 (0.54)	4.29 (0.59)	
PEOU_score				0.6
Median (Min - Max)	4.00 (1.00–5.00)	4.00 (1.00–5.00)	4.00 (1.00–5.00)	
Mean (SD)	3.89 (0.99)	3.92 (0.96)	3.84 (1.08)	
PA_score				**0**.**015**
Median (Min - Max)	4.00 (2.00–5.00)	4.00 (2.00–5.00)	4.00 (2.00–5.00)	
Mean (SD)	4.32 (0.57)	4.35 (0.54)	4.23 (0.63)	
PR_score				0.4
Median (Min - Max)	4.00 (1.00–5.00)	4.00 (1.00–5.00)	4.00 (1.00–5.00)	
Mean (SD)	3.26 (1.14)	3.28 (1.13)	3.22 (1.14)	
FC_score				0.8
Median (Min - Max)	4.00 (1.00–5.00)	4.00 (1.00–5.00)	4.00 (1.00–5.00)	
Mean (SD)	3.69 (1.05)	3.68 (1.07)	3.72 (1.02)	
PE_score				0.071
Median (Min - Max)	4.00 (1.00–5.00)	4.00 (1.00–5.00)	4.00 (2.00–5.00)	
Mean (SD)	4.28 (0.76)	4.29 (0.81)	4.27 (0.62)	
SN_score				**<0**.**001**
Median (Min - Max)	4.00 (1.00–5.00)	4.00 (1.00–5.00)	4.00 (1.00–5.00)	
Mean (SD)	3.96 (0.90)	4.05 (0.88)	3.74 (0.93)	
AT_score				0.5
Median (Min - Max)	5.00 (1.00–5.00)	5.00 (1.00–5.00)	5.00 (1.00–5.00)	
Mean (SD)	4.51 (0.61)	4.53 (0.57)	4.46 (0.69)	
ITU_score				0.5
Median (Min - Max)	4.00 (1.00–5.00)	4.00 (1.00–5.00)	4.00 (2.00–5.00)	
Mean (SD)	4.34 (0.68)	4.34 (0.69)	4.33 (0.64)	
AU_score				**<0**.**001**
Median (Min - Max)	4.00 (1.00–5.00)	4.00 (1.00–5.00)	4.00 (1.00–5.00)	
Mean (SD)	4.25 (0.69)	4.33 (0.63)	4.06 (0.81)	

Values in bold indicate statistical significance at *p* < 0.05.

^a^ Wilcoxon rank sum test.

### 4.4 Analysis per question of constructs and qualification

The results are displayed in Supplementary File 3.

#### 4.4.1 Perceived usefulness

Regarding questions PU_1 “mHealth.ci improves the quality of care provided by CHWs.”, and PU_2 “mHealth.ci helps monitor community health status better”, we observed a statistically significant difference (*p* = 0.003) with a stronger conviction of the CHWs.

#### 4.4.2 Perceived ease of use (PEOU)

***F***or question PEOU_8 “I need a little time to get used to using mHealth.ci”, a higher proportion of FHWs felt they needed more time to get used to the platform (*p* = 0.012) compared to the CHWs.

#### 4.4.3 Context and environment (FC)

Regarding question FC_17 “Is the training received on mHealth.ci sufficient?”, CHWs expressed a much higher level of disagreement regarding the sufficiency of training (*p* < 0.001).

#### 4.4.4 Perceived advantage (PA)

A significant difference was observed in questions PA_9 “mHealth.ci saves me time in my work” and PA_11 “I think mHealth.ci increases accessibility to healthcare” between CHWs and FHWs. CHWs perceive the advantages of mHealth.ci in terms of team savings (*p* = 0.002) and accessibility to care (*p* = 0.018) better than FHWs.

#### 4.4.5 Perceived disadvantages (PR)

Regarding question PR_13 “I have concerns about data security on mHealth.ci” More CHWs think there are no data security issues (*p* = 0.010).

#### 4.4.6 Personal emotion (PE)

A significant difference in personal emotion is observed between the two groups across all questions: PE_18 “I'm satisfied with using mHealth”, PE_19 “Using mHealth makes me confident in my professional skills”, and PE_20 “I feel stressed about using mHealth”.

More FHWs than CHWs declared that they are not satisfied with using mHealth.ci (*p* = 0.007). For more CHWs than FHWs stated that using the platform makes them feel confident in their professional skills (*p* = 0.005). More CHWs than FHWs mentioned that they feel stress when using the platform (*p* = 0.016).

#### 4.4.7 Social influence (SN)

A significant difference in social influence is observed between the two groups for questions SN_22 “I feel encouraged by my peers to use mHealth.ci” and SN_23 “My supervisors' recommendations influence my use of mHealth.ci”. CHWs feel more encouraged by their peers than FHWs (*p* < 0.001). More FHWs disagree with the influence of supervisors on their use of mHealth.ci (*p* < 0.001).

#### 4.4.8 Attitude (ATT)

A significant difference in social influence is observed between the two groups for questions ATT_24 “I have a positive attitude towards using mHealth.ci” and ATT_26 “I prefer to use mHealth.ci rather than paper-based system.”

We observed that CHWs seem to have a more positive attitude than FHWs (*p* = 0.032). We noted more neutral opinions among FHWs than CHWs on the preference between the two methods (*p* = 0.047).

#### 4.4.9 Actual use (AU)

A significant difference in personal emotion is observed between the two groups across all questions: AU_30 “I use mHealth.ci regularly for my work.”, AU_31 “I've already integrated mHealth.ci into my daily practices”, AU_32 “I feel comfortable using mHealth.ci in critical situations.”

CHWs reported having integrated mHealth.ci into their daily routines than FHWs (*p* = 0.014). They reported using mHealth.ci more regularly than FHWs (*p* = 0.019) and they felt more comfortable using mHealth.ci in critical situations (*p* = 0.020).

## 5 Discussion

Globally, the use of mobile health in community health information systems is increasing. Numerous studies have attempted to assess the acceptability of digital health technologies such as mobile health, and to identify critical success factors for the implementation of these digital health technologies in various contexts (2, 5–7, 9–11, 15–17). To the authors' knowledge, this is the first study carried out in West Africa to compare acceptability of mHealth.ci across two categories of end-users: the frontline health workers and the community health workers. The purpose of the study is to improve the adoption and implementation of mHealth.ci.

The findings showed in general a good level of acceptance of mHealth.ci among end-users. Two-thirds of them rated the tool as “totally acceptable” indicating a full acceptance. The remaining third found the tool “acceptable” the tool meaning that they have accepted the technology but maintain certain reservations regarding its implementation or functionality.

A large proportion of end-users had no prior experience of mobile healthcare applications, highlighting a notable lack of digital literacy among the target population. Digital literacy of health workers is critical to properly implement digital technologies in the health system. A study conducted in Ethiopia among healthcare providers in the public centers showed that their basic digital competency is low. This study reported that the characteristics of health workers (sex, educational status, profession type, monthly income, and years of experience) are predictors of digital literacy (18). In the study, CHWs had less experience with mobile health applications. These disparities are critical as they can profoundly influence the approach to implement mHealth.ci. For instance, less prior experience with mobile applications among CHWs could necessitate more extensive and tailored training.

Even though others have suggested that health workers with low preexisting digital literacy may have difficulties transitioning to mHealth technologies and be associated with lower acceptability and perceptions of its usefulness (19), our study highlighted the ability to introduce a mHealth app to a population of community health workers with low previous experience with mobile health. Despite their lower level of digital literacy and education than FHWs, CHWs reported a positive attitude and a greater acceptance. In addition, they recognized the usefulness and benefits of mHealth.ci and they seem to have integrated the platform more into their routine. This could be attributed to the nature of their work, where a mobile health application could offer more immediate and tangible benefits in community environments compared to a paper-based system. Our results are in line with another study that showed mHealth adoption among healthcare workers with little previous experience of smartphones. According to this study the use of pictograms to illustrate symptoms and the provision of training in the local language facilitated adoption of the mHealth app among FHWs and CHWs (20). Another study conducted in India CHWs reported that mHealth improved their performance and ability to perform tasks by supporting the ability to adhere to protocols (e.g., adherence to the correct number of required ANC visits) and ability to deliver consistent information and this has been reported in this study (21). In our study, CHWs reported both increased confidence and heightened stress while using the mHealth.ci platform. This dual emotional response can be interpreted through Cognitive Load Theory, which suggests that the mental effort required to learn new digital tools can temporarily overwhelm users, particularly those with limited prior experience (22). From a behavioral adaptation perspective, this stress may reflect the transitional strain of incorporating new workflows, even when the tool is perceived as beneficial. These findings underscore the need for training that reduces unnecessary complexity and supports gradual user adaptation. These findings also add a nuanced emotional dimension to the Louart S. framework, which considers “Personal Emotion” (15). Indeed, “stress” is often considered as a negative predictor of rejection. Our study suggests a “constructive stress” in digital health technology learning, where the pressure of learning coexists with an increased sense of confidence and desire to learn.

Moreover, CHWs expressed a much higher level of disagreement regarding the sufficiency of training. Given their lower prior experience with mobile applications, this strongly suggests that the training provided was inadequate for CHWs. This has direct implications for future training programs, emphasizing the need for more comprehensive and hands-on training tailored to their specific needs and digital literacy levels. On the other hand, a higher proportion of FHWs were not satisfied with using mHealth.ci and felt they needed more time to get used to it, despite their higher education and mobile application experience. This contrast could be explained by the fact that the FHWs need to be familiar with their platform and that of the CHWs they supervise, which could constitute a workload. In addition, our findings provide a significant counterpoint to the TAM framework which often suggests that higher education and prior experience with digital technology result in higher ease of use and acceptance (14).

The findings also showed that peer support is important for CHWs and highlighted the potential of leveraging peer networks for successful adoption and continued use among this group. Findings from India and Indonesia reported that support from peers, supervisors and communities strengthened CHWs' intention to adopt mHealth tools, in line with social influence theory (23–25). A study conducted in Uganda showed that a good collaboration and communication among key stakeholders facilitate mHealth programs (7). The same study in Uganda showed that poor working relationships with supervisors functioned as a barrier to mHealth. In our study, more FHWs disagreed with the influence of supervisors on their use of mHealth.ci, suggesting that top-down directives might be less effective for FHWs or that FHWs have more autonomy in their technology use decisions. These findings underscore the value of acceptance models in identifying early user responses. While the results demonstrate a high level of short-term acceptance, it is essential to distinguish these initial perceptions from long-term adoption. These disparities observed among CHWs and FHWs highlight that while “end-user acceptance” is a primary driver, it is not a static or sufficient condition for sustainable digital health implementation. The transition from acceptance to long-term digital adoption will require more than favorable user perceptions, including supportive supervision, adequate training (i.e., local language training, formalization of peer support), and adaptive change management strategies embedded within the health system such as strong leadership, clear communication, comprehensive training, stakeholder engagement, financial and non-financial incentives (16, 26–29). Moreover, regarding national scale-up of mHealth.ci, decision-makers should adopt a phased approach rather than a rapid nationwide rollout. This incremental approach enables the stabilization of infrastructure (electricity and network) and allows for platform adjustments based on early feedback from pilot health regions like Poro.

### 5.1 Study limitations

This study presents interesting results but has certain limitations that should be taken into consideration. First, data collection coincided with national vaccination days, resulting in the involvement of numerous end-users in related activities. Although our sample is representative of the end-user population, a larger sample could have captured a greater diversity of opinions with more accurate estimates and therefore a lower margin of error. In addition, these findings are subjective perceptions, and we acknowledge the possibility of social desirability and self-report bias on the part of end-users when answering questions. Because data were collected through face-to-face interviews using a structured questionnaire, there is an inherent risk of social desirability bias. Participants, particularly CHWs whose work is closely monitored, may have felt inclined to provide positive feedback about a government-mandated digital platform. However, we found a certain consistency in the responses given. Furthermore, effect size is crucial for interpreting the real-world importance of our findings. In the context of implementation science, even small effects can be significant. For instance, the small effect for actual use (Cliff's *δ* = 0.17), even one-month post-implementation, suggests a meaningful head start in integration for CHWs that, if sustained, could lead to larger impacts over time. Finally, the sociodemographic differences between groups, although documented, were not statistically controlled for in the bivariate analyses. There is therefore a risk of residual confounding in interpreting the observed differences. The ongoing multivariate analyses will allow more precise quantification of the independent effect of sociodemographic characteristics. Despite these limitations, this study makes an important contribution by documenting, for the first time in this context, the initial perceptions of two categories of health workers facing a digital innovation.

## 6 Conclusion

The study findings provide critical insight into the mHealth.ci acceptability among two categories of end-users. CHWs, despite limited digital literacy and mobile app experience, expressed strong enthusiasm and reported having integrated mHealth.ci in their routine. FHWs, who had higher digital proficiency, were less satisfied and took longer to be familiar with the platform. These results also highlighted the need to adopt effective change management strategies and a structured, adaptable plan to minimize resistance and ensure long-term integration. Moreover, while this study focuses on the Ivorian context, the observed impact of digital literacy on tool acceptance highlights a critical area for global health research. Future cross-country collaborations could provide a more comprehensive understanding of these determinants, facilitating knowledge exchange necessary for the global scaling of mHealth platform.

## Data Availability

The original contributions presented in the study are included in the article/Supplementary Material, further inquiries can be directed to the corresponding author.
