# Supplementary material for: Differential acceptance of a national digital health platform among community and frontline health workers in Cote d'Ivoire: a cross-sectional study
Source: Front Digit Health. 2026 May 4;8:1785017. doi: 10.3389/fdgth.2026.1785017 (PMC13180953; doi:10.3389/fdgth.2026.1785017)
Supplement: Supplementary file 1 [file Supplementaryfile1.pdf]

# mHealth End User Acceptance questionnaire

## 1. ADMINISTRATIVE INFORMATION

- Survey Date : _________________
- District Code :
  - [ ] Korhogo 1
  - [ ] Dikodougou
  - [ ] Sinematiali
  - [ ] M'bengue
  - [ ] Korhogo 2
- Health Facility Code: _________________
- Geographic Zone:
  - [ ] Urban
  - [ ] Rural
- Interviewer Code:
- Interviewee Position Number (1-10) : _________________
- Health Worker ID Number : _________________
- Did the interviewee give informed consent?
  - [ ] Yes
  - [ ] No
- If refusal, specify reason (or "N/A"): _________________

---

## 2. End user characteristics

- **Qualification:**
  1. [ ] Midwife / Accoucheur
  2. [ ] State Registered Nurse
  3. [ ] Community Health Worker (CHW)
  4. [ ] Other (please specify): _________________
- **Years of Experience:**
  - [ ] Less than 1 year
  - [ ] 1 to 4 years
  - [ ] 5 to 9 years
  - [ ] 10 years and more
- **Education Level:**
  - [ ] No formal schooling
  - [ ] Primary
  - [ ] Secondary
  - [ ] University
- **Demographics:**
  - **Date of Birth:** _________________
  - **Age at last birthday:** _________________
  - **Sex:** [ ] Male [ ] Female
- **Previous experience with health-related mobile apps?**
  - [ ] Yes
  - [ ] No

## 3.

PERCEPTIONS AND ATTITUDES
Please rate the following statements on a scale of 1 to 5:

*(1 = Strongly Disagree, 2 = Disagree, 3 = Neutral, 4 = Agree, 5 = Strongly Agree)*
49494949

| # | Domain & Statement | 1 | 2 | 3 | 4 | 5 |
|---|---|---|---|---|---|---|
| | **Domain 1: Perceived usefulness** | | | | | |
| 1 | mHealth.ci improves the quality of care provided by CHWs. | [ ] | [ ] | [ ] | [ ] | [ ] |
| 2 | mHealth.ci improves data quality. | [ ] | [ ] | [ ] | [ ] | [ ] |
| 3 | mHealth.ci helps me perform tasks more efficiently. | [ ] | [ ] | [ ] | [ ] | [ ] |
| 4 | mHealth.ci improves the completeness/timeliness of reports. | [ ] | [ ] | [ ] | [ ] | [ ] |
| 5 | mHealth.ci helps monitor community health status better. | [ ] | [ ] | [ ] | [ ] | [ ] |
| | **Domain 2: Perceived Ease of Use** [56] | | | | | |
| 6 | I find mHealth.ci easy to use. | [ ] | [ ] | [ ] | [ ] | [ ] |
| 7 | The features of mHealth.ci are intuitive. | [ ] | [ ] | [ ] | [ ] | [ ] |
| 8 | I need little time to get used to mHealth.ci. | [ ] | [ ] | [ ] | [ ] | [ ] |
| | **Domain 3: Perceived Advantages** | | | | | |
| 9 | mHealth.ci saves me time in my work. | [ ] | [ ] | [ ] | [ ] | [ ] |
| 10 | mHealth.ci allows quick access to essential information. | [ ] | [ ] | [ ] | [ ] | [ ] |
| 11 | mHealth.ci increases accessibility to healthcare. | [ ] | [ ] | [ ] | [ ] | [ ] |
| | **Domain 4: Perceived Disadvantages** | | | | | |
| 12 | mHealth.ci presents technical difficulties. | [ ] | [ ] | [ ] | [ ] | [ ] |
| 13 | I have concerns about data security on mHealth.ci. | [ ] | [ ] | [ ] | [ ] | [ ] |
| 14 | Using mHealth.ci can be confusing at times. | [ ] | [ ] | [ ] | [ ] | [ ] |
| | **Domain 5: Context & Environment** | | | | | |
| 15 | My work environment facilitates mHealth.ci use. | [ ] | [ ] | [ ] | [ ] | [ ] |
| 16 | Available resources facilitate the use of mHealth.ci. | [ ] | [ ] | [ ] | [ ] | [ ] |
| 17 | The training received on mHealth.ci is sufficient. | [ ] | [ ] | [ ] | [ ] | [ ] |
| | **Domain 6: Personal Emotion** | | | | | |
| 18 | I feel satisfied using mHealth.ci. | [ ] | [ ] | [ ] | [ ] | [ ] |
| 19 | mHealth.ci makes me feel confident in my skills. | [ ] | [ ] | [ ] | [ ] | [ ] |
| 20 | I feel stressed about using mHealth.ci. | [ ] | [ ] | [ ] | [ ] | [ ] |
| | **Domain 7: Social Influence** | | | | | |
| 21 | My colleagues use mHealth.ci. | [ ] | [ ] | [ ] | [ ] | [ ] |
| 22 | I feel encouraged by my peers to use mHealth.ci. | [ ] | [ ] | [ ] | [ ] | [ ] |
| 23 | Supervisor recommendations influence my usage. | [ ] | [ ] | [ ] | [ ] | [ ] |

| # | Domain & Statement | 1 | 2 | 3 | 4 | 5 |
|---|---|---|---|---|---|---|
| | **Domain 8: Attitude toward mHealth.ci** | | | | | |
| 24 | I have a positive attitude toward mHealth.ci. | [ ] | [ ] | [ ] | [ ] | [ ] |
| 25 | It is an important advancement for community health. | [ ] | [ ] | [ ] | [ ] | [ ] |
| 26 | I prefer mHealth.ci over old methods. | [ ] | [ ] | [ ] | [ ] | [ ] |
| | **Domain 9 : Intention to Use** | | | | | |
| 27 | I plan to use mHealth.ci in the future. | [ ] | [ ] | [ ] | [ ] | [ ] |
| 28 | I am likely to try new mHealth.ci features. | [ ] | [ ] | [ ] | [ ] | [ ] |
| 29 | I would recommend mHealth.ci to colleagues. | [ ] | [ ] | [ ] | [ ] | [ ] |
| | **Domain 10 : Actual use** | | | | | |
| 30 | I regularly use mHealth.ci tools in my work. | [ ] | [ ] | [ ] | [ ] | [ ] |
| 31 | I have integrated mHealth.ci into daily practices. | [ ] | [ ] | [ ] | [ ] | [ ] |
| 32 | I feel comfortable using it in critical situations. | [ ] | [ ] | [ ] | [ ] | [ ] |

---

## 4. OVERALL ACCEPTABILITY

33. Generally, how would you rate your level of acceptability of the mHealth.ci digital solution?

- [ ] 1 = Totally Unacceptable
- [ ] 2 = Unacceptable
- [ ] 3 = Neutral
- [ ] 4 = Acceptable
- [ ] 5 = Totally Acceptable

---

## 5. TECHNICAL DATA

- Latitude (x.y°): __________________
- Longitude (x.y°): __________________
- Altitude (m): __________________
- Accuracy (m): __________________
- Observations: __________________

---
