# Supplementary material for: Differential acceptance of a national digital health platform among community and frontline health workers in Cote d'Ivoire: a cross-sectional study
Source: Front Digit Health. 2026 May 4;8:1785017. doi: 10.3389/fdgth.2026.1785017 (PMC13180953; doi:10.3389/fdgth.2026.1785017)
Supplement: Supplementary file 2 [file Supplementaryfile2.pdf]

Supplementary file 2: Internal Consistency Analysis of Research Constructs (Cronbach's Alpha)

**Construct** <chr>	**Number of Items**	**Alpha Crombach Reliability**	**95% CI Lower** <dbl>	**95% CI Upper** <dbl>	**Interpretation** <chr>
PU	5	0.80	0.785	0.910	Good
PEOU	3	0.67	0.569	0.745	Questionable
PA	3	0.66	0.573	0.736	Questionable
PR	3	0.60	0.494	0.689	Questionable
FC	3	0.59	0.441	0.635	Poor
PE	3	0.71	0.588	0.803	Acceptable
SN	3	0.61	0.523	0.701	Questionnable
AT	3	0.70	0.535	0.800	Acceptable
ITU	3	0.75	0.684	0.805	Acceptable
AU	3	0.76	0.681	0.826	Acceptable
