# Supplementary material for: Differential acceptance of a national digital health platform among community and frontline health workers in Cote d'Ivoire: a cross-sectional study
Source: Front Digit Health. 2026 May 4;8:1785017. doi: 10.3389/fdgth.2026.1785017 (PMC13180953; doi:10.3389/fdgth.2026.1785017)
Supplement: Supplementary file 3 [file Supplementaryfile3.pdf]

Supplementary file 3: Differential acceptance by questions of domain among end-users

**Perceived usefulness (PU)**	**Overall** N = 280[1]	CHW N = 200[1]	FWH N = 80[1]	**p-value**[2]
**QPU_1. mHealth.ci improves the quality of care provided by CHWs.**				0.003
2 = disagree	0.36% (1/280)	0.00% (0/200)	1.25% (1/80)	
3 = neutral	1.07% (3/280)	1.00% (2/200)	1.25% (1/80)	
4 = agree	62.14% (174/280)	57.00% (114/200)	75.00% (60/80)	
5 = strongly disagree	36.43% (102/280)	42.00% (84/200)	22.50% (18/80)	
**QPU_2. mHealth.ci improves data quality.**				0.5
2 = disagree	1.07% (3/280)	0.50% (1/200)	2.50% (2/80)	
3 = neutral	1.07% (3/280)	1.00% (2/200)	1.25% (1/80)	
4 = agree	54.64% (153/280)	54.50% (109/200)	55.00% (44/80)	
5 = strongly disagree	43.21% (121/280)	44.00% (88/200)	41.25% (33/80)	
**QPU_3. mHealth.ci helps me perform tasks more efficiently.**				0.13
2 = disagree	0.71% (2/280)	0.50% (1/200)	1.25% (1/80)	
3 = neutral	1.07% (3/280)	0.50% (1/200)	2.50% (2/80)	
4 = agree	53.21% (149/280)	51.00% (102/200)	58.75% (47/80)	
5 = strongly agree	45.00% (126/280)	48.00% (96/200)	37.50% (30/80)	
**QPU_4. mHealth.ci improves the completeness/timeliness of reports.**				0.4
1 =strongly disagree	0.36% (1/280)	0.00% (0/200)	1.25% (1/80)	
2 = disagree	0.71% (2/280)	1.00% (2/200)	0.00% (0/80)	
3 = neutral	1.43% (4/280)	1.00% (2/200)	2.50% (2/80)	
4 = agree	55.36% (155/280)	55.50% (111/200)	55.00% (44/80)	
5 = strongly disagree	42.14% (118/280)	42.50% (85/200)	41.25% (33/80)	
**QPU_5. mHealth.ci helps monitor community health status better.**				0.003
2 = disagree	0.36% (1/280)	0.50% (1/200)	0.00% (0/80)	
3 = neutral	1.07% (3/280)	0.00% (0/200)	3.75% (3/80)	
4 = disagree	58.93% (165/280)	55.50% (111/200)	67.50% (54/80)	
5 = strongly agree	39.64% (111/280)	44.00% (88/200)	28.75% (23/80)	

[1]% (n/N)

[2]Fisher's exact test

**Perceived Ease of Use (PEOU)**	**Overall** N = 280[1]	**ASC** N = 200[1]	**IDE_SF** N = 80[1]	**p-value**[2]
**QPEOU_6. I find mHealth.ci easy to use.**				0.10
2 = disagree	8.21% (23/280)	10.00% (20/200)	3.75% (3/80)	
3 = neutral	2.50% (7/280)	1.50% (3/200)	5.00% (4/80)	
4 = agree	50.71% (142/280)	49.00% (98/200)	55.00% (44/80)	
5 = strongly agree	38.57% (108/280)	39.50% (79/200)	36.25% (29/80)	
**QPEOU_7. The features of mHealth.ci are intuitive.**				0.2
2 = disagree	1.79% (5/280)	1.00% (2/200)	3.75% (3/80)	
3 = neutral	8.93% (25/280)	8.50% (17/200)	10.00% (8/80)	
4 = agree	61.79% (173/280)	64.50% (129/200)	55.00% (44/80)	
5 = strongly agree	27.50% (77/280)	26.00% (52/200)	31.25% (25/80)	
**QPEOU_8. I need little time to get used to mHealth.ci.**				0.012
1 = strongly disagree	7.50% (21/280)	6.00% (12/200)	11.25% (9/80)	
2 = disagree	24.64% (69/280)	22.50% (45/200)	30.00% (24/80)	
3 = neutral	5.36% (15/280)	4.50% (9/200)	7.50% (6/80)	
4 = agree	52.14% (146/280)	58.50% (117/200)	36.25% (29/80)	
5 = strongly agree	10.36% (29/280)	8.50% (17/200)	15.00% (12/80)	

[1]% (n/N)

[2]Fisher's exact test

**Perceived Advantages (PA)**	**Overall** N = 280[1]	**ASC** N = 200[1]	**IDE_SF** N = 80[1]	**p-value**[2]
**QPA_9. mHealth.ci saves me time in my work**				0.002
2 = disagree	1.79% (5/280)	0.50% (1/200)	5.00% (4/80)	
3 = neutral	1.43% (4/280)	0.50% (1/200)	3.75% (3/80)	
4 = agree	57.14% (160/280)	55.00% (110/200)	62.50% (50/80)	
5 = strongly agree	39.64% (111/280)	44.00% (88/200)	28.75% (23/80)	
**QPA_10. mHealth.ci allows quick access to essential information.**				0.6
2 = disagree	0.36% (1/280)	0.50% (1/200)	0.00% (0/80)	
4 = agree	58.93% (165/280)	60.50% (121/200)	55.00% (44/80)	
5 = strongly agree	40.71% (114/280)	39.00% (78/200)	45.00% (36/80)	
**QPA_11. mHealth.ci increases accessibility to healthcare.**				0.018
2 = disagree	1.43% (4/280)	1.50% (3/200)	1.25% (1/80)	
3 = neutral	4.29% (12/280)	2.00% (4/200)	10.00% (8/80)	
4 = agree	66.43% (186/280)	66.00% (132/200)	67.50% (54/80)	
5 = strongly agree	27.86% (78/280)	30.50% (61/200)	21.25% (17/80)	

[1]% (n/N)

[2]Fisher's exact test

**Perceived disadvantages**	**Overall** N = 280[1]	**ASC** N = 200[1]	**IDE_SF** N = 80[1]	**p-value**[2]
**QPR_12. mHealth.ci presents technical difficulties.**				0.3
1 = strongly disagree	6.79% (19/280)	6.50% (13/200)	7.50% (6/80)	
2 = disagree	34.64% (97/280)	38.00% (76/200)	26.25% (21/80)	
3 = neutral	4.29% (12/280)	4.50% (9/200)	3.75% (3/80)	
4 = agree	45.00% (126/280)	43.50% (87/200)	48.75% (39/80)	
5 = strongly agree	9.29% (26/280)	7.50% (15/200)	13.75% (11/80)	
**QPR_13. I have concerns about data security on mHealth.ci.**				0.010
1 = strongly disagree	11.79% (33/280)	11.00% (22/200)	13.75% (11/80)	
2 = disagree	53.21% (149/280)	56.00% (112/200)	46.25% (37/80)	
3 = neutre	13.93% (39/280)	10.00% (20/200)	23.75% (19/80)	
4 = agree	17.50% (49/280)	18.00% (36/200)	16.25% (13/80)	
5 = strongly agree	3.57% (10/280)	5.00% (10/200)	0.00% (0/80)	
**QPR_14. Using mHealth.ci can be confusing at times.**				0.12
1 =strongly disagree	10.71% (30/280)	10.50% (21/200)	11.25% (9/80)	
2 = disagree	52.86% (148/280)	54.00% (108/200)	50.00% (40/80)	
3 = neutral	6.43% (18/280)	4.00% (8/200)	12.50% (10/80)	
4 = agree	27.86% (78/280)	29.50% (59/200)	23.75% (19/80)	
5 = strongly agree	2.14% (6/280)	2.00% (4/200)	2.50% (2/80)	

[1]% (n/N)

[2]Fisher's exact test

**Context and Environnement**	**Overall** N = 280[1]	**ASC** N = 200[1]	**IDE_SF** N = 80[1]	**p-value**[2]
**QFC_15. My work environment facilitates mHealth.ci use**				0.2
1 = strongly disagree	0.71% (2/280)	0.50% (1/200)	1.25% (1/80)	
2 = disagree	12.14% (34/280)	9.50% (19/200)	18.75% (15/80)	
3 = neutral	2.50% (7/280)	2.50% (5/200)	2.50% (2/80)	
4 = agree	62.50% (175/280)	64.00% (128/200)	58.75% (47/80)	
5 = strongly agree	22.14% (62/280)	23.50% (47/200)	18.75% (15/80)	
**QFC_16. Available resources facilitate the use of mHealth.ci**				0.7
1 = strongly disagree	2.14% (6/280)	2.00% (4/200)	2.50% (2/80)	
2 = disagree	11.07% (31/280)	9.50% (19/200)	15.00% (12/80)	
3 = neutral	4.29% (12/280)	4.50% (9/200)	3.75% (3/80)	
4 = agree	67.50% (189/280)	69.00% (138/200)	63.75% (51/80)	
5 = strongly agree	15.00% (42/280)	15.00% (30/200)	15.00% (12/80)	
**QFC_17. The training received on mHealth.ci is sufficient.**				<0.001
1 = strongly disagree	4.64% (13/280)	5.00% (10/200)	3.75% (3/80)	
2 = disagree	34.29% (96/280)	41.50% (83/200)	16.25% (13/80)	
3 = neutral	2.50% (7/280)	1.00% (2/200)	6.25% (5/80)	
4 = agree	42.14% (118/280)	36.50% (73/200)	56.25% (45/80)	
5 = strongly agree	16.43% (46/280)	16.00% (32/200)	17.50% (14/80)	

[1]% (n/N)

[2]Fisher's exact test

**Personal Emotion**	**Overall** N = 280[1]	**ASC** N = 200[1]	**IDE_SF** N = 80[1]	**p-value**[2]
**QPE_18. I feel satisfied using mHealth.ci**				0.007
1 = strongly disagree	0.36% (1/280)	0.50% (1/200)	0.00% (0/80)	
2 = disagree	2.14% (6/280)	1.50% (3/200)	3.75% (3/80)	
3 = neutral	0.36% (1/280)	0.50% (1/200)	0.00% (0/80)	
4 = agree	48.93% (137/280)	43.50% (87/200)	62.50% (50/80)	
5 = strongly agree	48.21% (135/280)	54.00% (108/200)	33.75% (27/80)	
**QPE_19. mHealth.ci makes me feel confident in my skills**				0.005
2 = disagree	0.36% (1/280)	0.00% (0/200)	1.25% (1/80)	
3 = neutral	1.43% (4/280)	1.00% (2/200)	2.50% (2/80)	
4 = agree	60.00% (168/280)	55.50% (111/200)	71.25% (57/80)	
5 = strongly disagree	38.21% (107/280)	43.50% (87/200)	25.00% (20/80)	
**QPE_20. I feel stressed about using mHealth.ci.**				0.016
1 = strongly disagree	35.00% (98/280)	32.00% (64/200)	42.50% (34/80)	
2 = disagree	50.00% (140/280)	49.50% (99/200)	51.25% (41/80)	
3 = neutral	2.86% (8/280)	2.50% (5/200)	3.75% (3/80)	
4 = agree	10.36% (29/280)	13.50% (27/200)	2.50% (2/80)	
5 = strongly agree	1.79% (5/280)	2.50% (5/200)	0.00% (0/80)	

[1]% (n/N)

[2]Fisher's exact test

**Social influence**	**Overall** N = 280[1]	**ASC** N = 200[1]	**IDE_SF** N = 80[1]	**p-value**[2]
**QSN_21. My colleagues use mHealth.ci.**				0.5
2 = disagree	0.71% (2/280)	0.50% (1/200)	1.25% (1/80)	
3 = neutral	0.71% (2/280)	0.50% (1/200)	1.25% (1/80)	
4 = agree	73.93% (207/280)	73.50% (147/200)	75.00% (60/80)	
5 = strongly agree	24.64% (69/280)	25.50% (51/200)	22.50% (18/80)	
**QSN_22. I feel encouraged by my peers to use mHealth.ci**				<0.001
1 =strongly disagree	0.36% (1/280)	0.50% (1/200)	0.00% (0/80)	
2 = disagree	1.79% (5/280)	0.50% (1/200)	5.00% (4/80)	
3 = neutral	2.14% (6/280)	0.50% (1/200)	6.25% (5/80)	
4 = agree	61.07% (171/280)	59.00% (118/200)	66.25% (53/80)	
5 = strongly agree	34.64% (97/280)	39.50% (79/200)	22.50% (18/80)	
**QSN_23. Supervisor recommendations influence my usage**				<0.001
1 = strongly disagree	2.86% (8/280)	3.50% (7/200)	1.25% (1/80)	
2 = disagree	29.29% (82/280)	24.00% (48/200)	42.50% (34/80)	
3 = neutral	11.43% (32/280)	9.50% (19/200)	16.25% (13/80)	
4 = agree	40.36% (113/280)	41.50% (83/200)	37.50% (30/80)	
5 = strongly agree	16.07% (45/280)	21.50% (43/200)	2.50% (2/80)	

[1]% (n/N)
[2]Fisher's exact test

Attitude	**Overall** N = 280[1]	**ASC** N = 200[1]	**IDE_SF** N = 80[1]	**p-value**[2]
**QATT_24. I have a positive attitude toward mHealth.ci.**				0.032
1 =strongly disagree	0.36% (1/280)	0.50% (1/200)	0.00% (0/80)	
2 = disagree	1.43% (4/280)	0.00% (0/200)	5.00% (4/80)	
3 = neutral	2.14% (6/280)	2.50% (5/200)	1.25% (1/80)	
4 = agree	66.79% (187/280)	67.00% (134/200)	66.25% (53/80)	
5 = strongly agree	29.29% (82/280)	30.00% (60/200)	27.50% (22/80)	
**QATT_25. It is an important advancement for community health.**				0.6
1 =strongly disagree	0.36% (1/280)	0.50% (1/200)	0.00% (0/80)	
3 = neutral	0.36% (1/280)	0.50% (1/200)	0.00% (0/80)	
4 = agree	31.79% (89/280)	34.00% (68/200)	26.25% (21/80)	
5 = strongly agree	67.50% (189/280)	65.00% (130/200)	73.75% (59/80)	
**QATT_26. I prefer mHealth.ci over old methods.**				0.047
1 =strongly disagree	0.36% (1/280)	0.00% (0/200)	1.25% (1/80)	
2 = disagree	0.71% (2/280)	0.50% (1/200)	1.25% (1/80)	
3 = neutral	2.86% (8/280)	1.50% (3/200)	6.25% (5/80)	
4 = agree	27.86% (78/280)	27.00% (54/200)	30.00% (24/80)	
5 = strongly agree	68.21% (191/280)	71.00% (142/200)	61.25% (49/80)	

**Intention of use**	**Overall** N = 280[1]	**ASC** N = 200[1]	**IDE_SF** N = 80[1]	**p-value**[2]
**QITU_27. I plan to use mHealth.ci in the future**				0.081
1 = strongly disagree	0.36% (1/280)	0.50% (1/200)	0.00% (0/80)	
2 = disagree	0.36% (1/280)	0.50% (1/200)	0.00% (0/80)	
3 = neutral	1.79% (5/280)	0.50% (1/200)	5.00% (4/80)	
4 = agree	40.71% (114/280)	40.00% (80/200)	42.50% (34/80)	
5 = strongly agree	56.79% (159/280)	58.50% (117/200)	52.50% (42/80)	
**QITU_28. I am likely to try new mHealth.ci features**				0.8
1 = strongly disagree	1.07% (3/280)	1.50% (3/200)	0.00% (0/80)	
2 = disagree	5.36% (15/280)	6.00% (12/200)	3.75% (3/80)	
3 = neutral	5.00% (14/280)	5.50% (11/200)	3.75% (3/80)	
4 = agree	60.00% (168/280)	59.50% (119/200)	61.25% (49/80)	
5 = strongly agree	28.57% (80/280)	27.50% (55/200)	31.25% (25/80)	
**QITU_29. I would recommend mHealth.ci to colleagues**				0.061
2 = disagree	0.36% (1/280)	0.00% (0/200)	1.25% (1/80)	
3 = neutral	1.43% (4/280)	0.50% (1/200)	3.75% (3/80)	
4 = agree	57.50% (161/280)	57.50% (115/200)	57.50% (46/80)	
5 = strongly agree	40.71% (114/280)	42.00% (84/200)	37.50% (30/80)	

[1]% (n/N)

[2]Fisher's exact test

**Actual Use**	**Overall** N = 280[1]	**ASC** N = 200[1]	**IDE_SF** N = 80[1]	**p-value**[2]
**QAU_30. I regularly use mHealth.ci tools in my work**				0.019
2 = disagree	4.29% (12/280)	2.50% (5/200)	8.75% (7/80)	
3 = neutral	2.50% (7/280)	1.50% (3/200)	5.00% (4/80)	
4 = agree	55.36% (155/280)	55.00% (110/200)	56.25% (45/80)	
5 = strongly agree	37.86% (106/280)	41.00% (82/200)	30.00% (24/80)	
**QAU_31. I have integrated mHealth.ci into daily practices**				0.014
2 = disagree	2.14% (6/280)	1.00% (2/200)	5.00% (4/80)	
3 = neutral	1.79% (5/280)	1.00% (2/200)	3.75% (3/80)	
4 = agree	56.07% (157/280)	54.00% (108/200)	61.25% (49/80)	
5 = strongly agree	40.00% (112/280)	44.00% (88/200)	30.00% (24/80)	
**QAU_32. I feel comfortable using it in critical situations**				0.020
1 = strongly disagree	0.71% (2/280)	0.50% (1/200)	1.25% (1/80)	
2 = disagree	3.93% (11/280)	2.50% (5/200)	7.50% (6/80)	
3 = neutral	4.29% (12/280)	2.50% (5/200)	8.75% (7/80)	
4 = agree	62.50% (175/280)	63.50% (127/200)	60.00% (48/80)	
5 = strongly agree	28.57% (80/280)	31.00% (62/200)	22.50% (18/80)	

[1]% (n/N)

[2]Fisher's exact test
