# Supplementary figures and images for: Differential acceptance of a national digital health platform among community and frontline health workers in Cote d'Ivoire: a cross-sectional study
Source: Front Digit Health. 2026 May 4;8:1785017. doi: 10.3389/fdgth.2026.1785017 (PMC13180953; doi:10.3389/fdgth.2026.1785017)

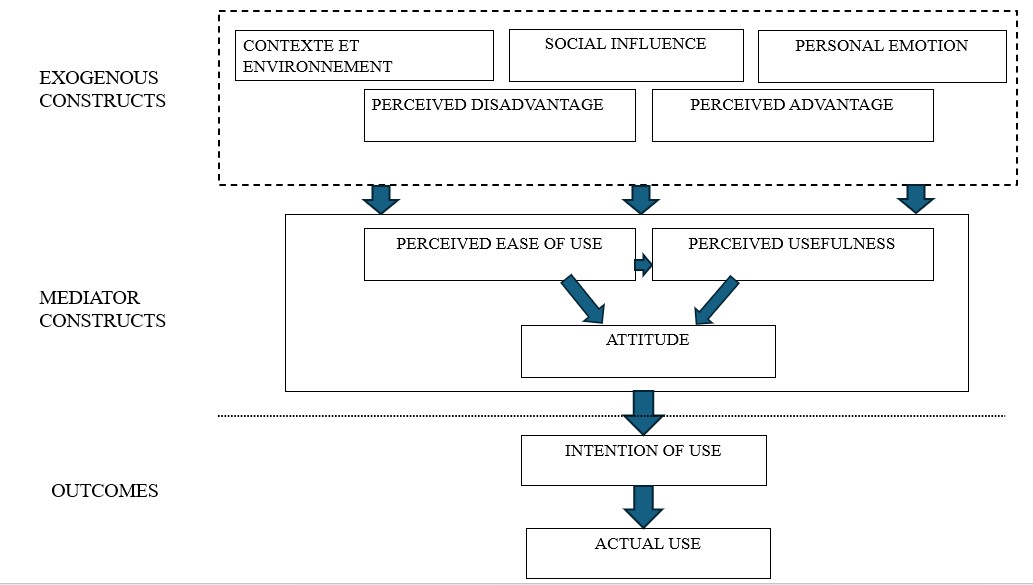

Supplement: Supplementary file 4 [file Image1.jpeg]
